# Evaluation the consistency of location of moist desquamation and skin high dose area for breast cancer patients receiving adjuvant radiotherapy after breast conservative surgery

**DOI:** 10.1186/1748-717X-8-50

**Published:** 2013-03-06

**Authors:** Li-Min Sun, Eng-Yen Huang, Ji-An Liang, Fan-Yun Meng, Gia-Hsin Chang, Min-Jen Tsao

**Affiliations:** 1 Department of Radiation Oncology, Zuoying Branch of Kaohsiung Armed Forces General Hospital, 553 Junxiao Rd, Zuoying District, Kaohsiung, Taiwan; 2 Department of Radiation Oncology, Kaohsiung Chang Gung Memorial Hospital, Chung Gung University College of Medicine, Kaohsiung, Taiwan; 3 School of Traditional Chinese Medicine, Chung Gung University College of Medicine, Kaohsiung, Taiwan; 4 Department of Radiation Therapy and Oncology, China Medical University Hospital, Taichung, Taiwan; 5 School of Medicine, China Medical University, Taichung, Taiwan; 6 Deparment of General Surgery, Zuoying Branch of Kaohsiung Armed Forces General Hospital, Kaohsiung, Taiwan

**Keywords:** Breast cancer, Radiotherapy, Radiation dermatitis, High dose area

## Abstract

**Background:**

To evaluate whether the location of moist desquamation matches high dose area for breast cancer patients receiving adjuvant radiotherapy (RT) after breast conservative surgery.

**Methods:**

One hundred and nine breast cancer patients were enrolled to this study. Their highest skin dose area (the hot spot) was estimated from the treatment planning. We divided the irradiated field into breast; sternal/parasternal; axillary; and inframammary fold areas. The location for moist desquamation was recorded to see if it matches the hot spot. We also analyzed other possible risk factors which may be related to the moist desquamation.

**Results:**

Forty-eight patients with 65 locations developed moist desquamation during the RT course. Patients with larger breast sizes and easy to sweat are two independent risk factors for moist desquamation. The distribution of moist desquamation occurred most in the axillary area. All nine patients with the hot spots located at the axillary area developed moist desquamation at the axillary area, and six out of seven patients with the hot spots located at the inframammary fold developed moist desquamation there. The majority of patients with moist desquamation over the breast or sternal/parasternal areas had the hot spots located at these areas.

**Conclusions:**

For a patient with moist desquamation, if a hot spot is located at the axillary or inframammary fold areas, it is very likely to have moist desquamation occur there. On the other hand, if moist desquamation occurs over the breast or sternal/parasternal areas, we can highly expect these two areas are also the hot spot locations.

## Background

Breast cancer is the most common type of cancer diagnosed among women in the United States, excluding skin cancer [1]. According to statistics of the Department of Health, Executive Yuan, R.O.C., it is also the most common malignancy among women in Taiwan since 1996, and its incidence rate has increased by 22.2% from 2001 to 2005 [2]. The age-adjusted incidence rate has reached 62.38 new cases per 100,000 in 2008 [2]. Nowadays, Breast-conserving surgery (BCS), followed by radiotherapy (RT), has become accepted as an appropriate treatment for women with early breast cancer [3]. The modern RT technique can effectively reduce the dose to the underlying lung and heart, and the related-sequelae in these organs can be minimized [4,5]. The skin, however, is close to the target volume and inevitably receives a high radiation dose.

Skin is relatively radiosensitive and tends to have different degrees of damage after certain doses of radiation [6]. Therefore; we can expect the appearance of radiation dermatitis in the RT field. Moist desquamation is common and will cause the discomfort of patients or the interruption of treatment [7,8]. Grade II acute radiation dermatitis means moderate to brisk erythema, patchy moist desquamation (mostly confined to skin folds and creases), and moderate edema. Grade III means moist desquamation other than skin folds and creases and bleeding induced by minor trauma or abrasion [9]. The current study analyzed the possible risk factors related to the occurrence of moist desquamation for breast cancer patients receiving RT after BCS. The skin irradiated dose can be reflected on the isodose curve distribution of RT treatment planning, and we were interested in investigating whether the highest skin dose area (the hot spot) can predict the location of moist desquamation appearance. Therefore we focused on the evaluation of compatibility of the hot spot and location of the moist desquamation.

## Methods

### Patients

Between June 2010 and November 2011, 109 consecutive female patients with breast cancer after BCS were referred to our department for adjuvant RT and enrolled into this prospective study. The eligibility criteria were that patients with pathologically proven breast carcinoma, curative intent for RT (no stage IV disease), not recurrence, and ability to raise their arms steadily with a cast holding during daily RT. There was no age limitation.

All patients’ demographic characteristics as well as other important data were recorded. They include the patient’s age, body mass index (BMI, calculated from the body weight and height), breast size [volume measured by the computed tomography (CT)], lesion side, pathological stage defined by the seventh edition of the AJCC Cancer Staging Manual [10], either subjective (self-reported by patient) or objective (observed by staff) judgment of easy to sweat or neither, skin color (light or dark), treatment season (at least half of treatment times were in May through September or not), adjuvant chemotherapy and hormone therapy.

### Immobilization and treatment planning

A customized chest mold was cast for each patient for immobilization and reproducibility during computed tomography (CT) simulation and each treatment process. The material used for the cast was the Aquaplast Thermoplastic which can be easily molded and conformed to the curvature of skin [11]. Patients received CT simulation by the Philips ACQSim CT simulator with the cast. The radiation oncologist then contoured the target on the planned CT sections according to the guidelines of International Commission of Radiation Units and Measurements Reports 50 and 62 [12,13]. The breast margins shown on each slice of CT images were also delineated to measure breast volume. The radiation physicist did the planning in the ADAC Pinnacle Treatment Planning System thereafter. The clinical target volume (CTV) included the ipsilateral breast and possible axillary LNs. The RT for internal mammary chain LNs and/or ipsilateral supraclavicular LNs were reserved to cases with higher risk of tumor recurrence in those areas. The planning target volume (PTV) was CTV with extension of 0.5 to 1 cm margins.

We used bilateral opposed tangential fields on megavoltage linear accelerators with three-dimensional conformal radiotherapy (3-D CRT) technique to cover the breast, axillary LNs (except for stage 0 cases) and possible ipsilateral internal mammary LNs. One AP field with a tilt of 10 to 15 degree to spare more larynx and upper esophagus was used to cover SCF when the ipsilateral supraclavicular LNs was the target. Treatment plans were designed to deliver the prescribed dose to the target volumes with consideration of normal tissue constraints. The prescribed dose for the PTV was 50.4 Gy in 28 fractions with 6 MV x-ray, and for selected cases with high risk factors [14], we added the surgical scar boost for additional 10.8 Gy in 6 fractions via electron beam. We observed the isodose distribution for each patient and recorded the maximum point dose location shown on the isodose distribution as the hot spot for the overlying skin.

### Treatment process

**Figure 1:**
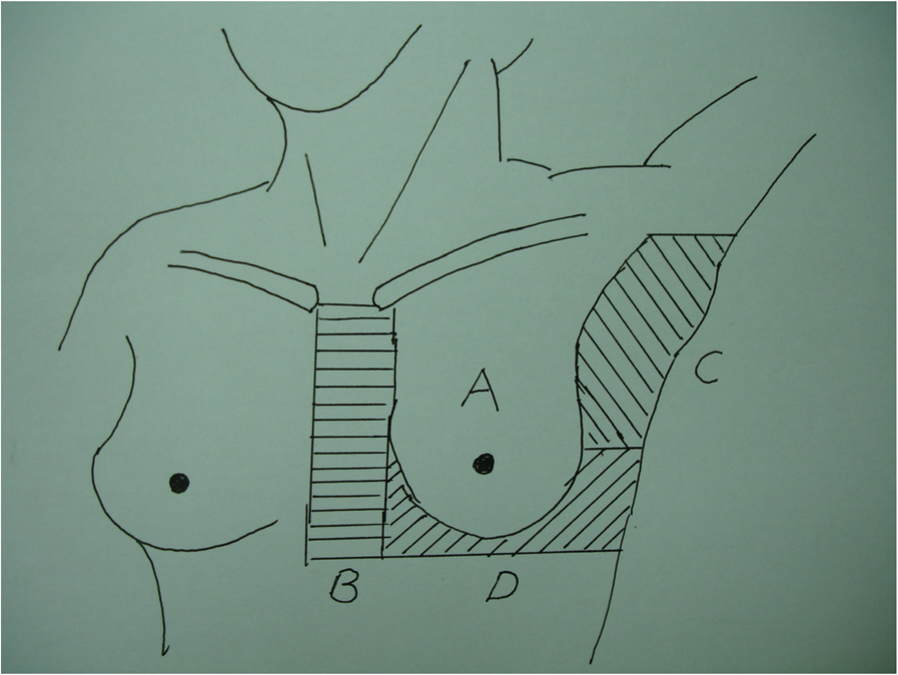
The 4 parts of irradiated area: A: main breast area; B: sternal/parasternal area; C: axillary area; D: inframammary area.

After 2 weeks of treatment, the corresponding physician and therapist started paying attention to observe if any moist desquamation occurs over patient’s skin, and they documented the location and RT dose when it occurred. Because not all patients received the electron boost for the surgical scar, we only recorded the skin reaction during the photon beam therapy (dose up to 50.4 Gy). We observed the acute skin reaction every treatment day between 3 and 6 weeks counted from the start of treatment. It’s inconvenient for us to objectively document the skin reaction after completion of RT, so we did not record the skin reaction thereafter. In order to conveniently document the hot spot area and the location of severe skin reaction, we have divided the RT area into 4 parts (Figure 1). There are: (A) main breast area, (B) sternal/ parasternal area, (C) axillary area, and (D) inframammary fold.

### Statistics

The chi-square test and logistic regression model were conducted for the univariate and multivariate analyses of the potential risk factors and moist desquamation, respectively. A p-value of less than 0.05 from a two-tailed test is considered statistically significant. All the biostatistics were performed by the software of Statistics with Stata (8th Edition, Brooks/Cole Inc., Belmont, CA).

## Results

Patient’s age ranged from 29 to 81 years old and median age was 51 years of age, and the majority was younger than 55 years of age. Breast size (represented by the volume which was measured from the CT planning system) ranged from 277 to 668 cc and median volume was 358 cc. The pathologic stage distribution is as below: stage 0: 22; stage I: 39; stage IIA: 16; stage IIB: 19; stage IIIA: 8; stage IIIB: 0; stage IIIC: 5. There were a total of 48 patients who developed moist desquamation at the dose ranged from 32.4 Gy to 50.4 Gy with the median dose of 45.0 Gy. Table 1 showed the results of univariate analysis for the relationship between the patient’s demographic and moist desquamation. Breast size and easy to sweat are the two factors with statistical significance. Patients with breast volume 350 cc or more or easy to sweat were prone to have moist desquamation. Larger BMI and treatment course in summer are supposed to have more occurrence of moist desquamation, and our data also showed this trend. However, the statistical analysis did not reach the significant level. Other factors did not show significant impact to predict moist desquamation, either. Further multivariate analysis did not change the results and demonstrated that the two predictors of moist desquamation are still patients with larger breast (p = 0.012) and easy to sweat (p = 0.007) (Table 2).

**Table 1:** Univariate analysis for the relationship between the patient’s characteristics and moist desquamation

**Parameters**	**Case number**	**Moist desquamation**			***P *value**
		**Yes**	**No**	**Percentage (%)**	
Age					0.523
< 55	65	27	38	41.5	
≧55	44	21	23	47.7	
BMI					0.181
< 25	51	19	32	37.3	
≧25	58	29	29	50.0	
Breast volume (cc)					0.004^*^
< 350	51	15	36	29.4	
≧350	58	33	25	56.9	
Side					0.492
Right	55	26	29	47.3	
Left	54	22	32	40.7	
Stage					0.469
0–I	61	25	36	41.0	
II–III	48	23	25	47.9	
Easy to sweat					0.001^*^
Yes	57	34	23	59.7	
No	52	14	38	26.9	
Skin color					0.243
Light	50	19	31	38.0	
Dark	59	29	30	49.2	
Summer					0.342
Yes	58	28	30	48.3	
No	51	20	31	39.2	
Chemotherapy					0.370
Yes	47	23	24	49.0	
No	62	25	37	40.3	
Hormone therapy					0.263
Yes	57	28	29	49.1	
No	52	20	32	38.5	

*Abbreviations:* BMI = Body mass index.

^*^Statistically significant.

**Table 2:** Multivariate analysis for the relationship between the patients’ characteristics and moist desquamation

**Parameters**	***P *value**
Age	0.758
BMI	0.904
Breast volume	0.012^*^
Side	0.583
Stage	0.330
Easy to sweat	0.007^*^
Skin color	0.618
Summer	0.225
Chemotherapy	0.240
Hormone therapy	0.550

*Abbreviations:* BMI = Body mass index.

^*^Statistically significant.

Table 3 illustrated the distribution of the hot spot locations and its relationship to area of moist desquamation. Each patient had one hot spot location of skin dose. The breast area is the most common location for the hot spot, followed by the axillary area, inframammary fold, and sterna/parasternal area. All nine patients with the skin hot spot located at axillary area developed moist desquamation at axillary area, and six out of seven patients with skin the hot spot located at inframammary fold developed moist desquamation exactly at inframammary fold. On the other hand, the use the hot spot to predict the moist desquamation location is not so expected for breast and sternal /parasternal areas.

**Table 3:** The distribution of the hot spot locations and its relationship to areas of moist desquamation

		**Moist desquamation occurred at the same area of the hot spot**	
**The hot spot location**	**# (total 48)**	**Yes (%)**	**No (%)**
Axillary	9	9 (100)	0 (0)
Breast	27	11 (40.7)	16 (59.3)
Inframammary fold	7	6 (85.7)	1 (14.3)
Sternal/parasternal area	5	3 (60.0)	2 (40.0)

A total of 65 locations of moist desquamation were observed in 48 patients. The distribution of the moist desquamation occurrence area and its relationship to the hot spot locations were shown in Table 4. The Axillary area is the most common site for the moist desquamation appearance, followed by the breast, inframammary fold, and sterna/parasternal area. The majority of patients with moist desquamation over the breast or sternal/parasternal areas had the hot spot located at these two areas. On the other hand, the occurrence of moist desquamation at the axillary area and inframammary fold are not so correlated with the hot spot location.

**Table 4:** The distribution of moist desquamation occurrence areas and its relationship to the hot spot locations

		**The hot spot located at that area**	
**Moist desquamation area**	**# (total 65)**	**Yes (%)**	**No (%)**
Axillary	35	9 (25.7)	26 (74.3)
Breast	14	11 (78.6)	3 (21.4)
Inframammary fold	12	6 (50.0)	6 (50.0)
Sternal/parasternal area	4	3 (75.0)	1 (25.0)

We also assessed the quality of treatment planning by several parameters: V95%, V105% and V110%. V95% is defined as the absolute volume (in cc) of PTV receiving 95% of the prescribed dose. V105% and V110% represent the absolute volume of PTV receiving 105% and 110% of the prescribed dose, respectively. Our data showed that the V95% ranged from 396 to 803 cc with a mean of 606 cc, the V105% ranged from 154 to 312 cc with a mean of 227 cc, and the V110% ranged from 67 to 148 cc with a mean of 103 cc.

## Discussion

This study found that those patients with larger breast size or easy to sweat are prone to get moist desquamation when they receive adjuvant RT after BCS for their breast cancer. The moist desquamation tends to occur at the axillary area and inframammary fold, especially when a hot spot of skin dose is also located at these areas.

The results from this study show the median age for our patients was 51 years. It is younger than the population-based data in the US which showed that the median age at diagnosis was 61 years of age from 2003 to 2007 [15]. In fact; breast cancer in Taiwan is characterized by a striking recent increase of incidence and a relatively young median age (45-49 years) at diagnosis. The westernization of lifestyle that is increasingly affecting younger generations of the Taiwanese may play an important role on this change [16].

The incidence of severe reaction is dependent on the total radiation dose, the dose per fraction, the overall treatment time, beam type and energy, and the surface area of the skin that is exposed to radiation [17]. Our data revealed that 48 out of 109 patients (44.0%) developed moist desquamation. This high rate is not unexpected and is compatible with prior studies [7,18]. Although appropriate management of moist desquamation will not leave obvious sequelae, the acute morbidity definitely impact a patient’s quality of life. Several ways to reduce skin toxicities either by using skin sparing techniques [19,20], or local applying of prophylactic medication are under investigation [21,22].

Our data found patients with breast volume 350-cc or larger had a significant higher risk to develop moist desquamation. It is compatible with earlier studies which showed larger breast size and brassiere size which has been linked to the risk of acute skin reactions [23-25], and this may relate to the overall treatment port size required. In addition, larger breasts which tend to fold over onto the chest are more predisposed to have severer skin reaction. BMI are supposed to be correlated to breast size, and our data also support it (p value < 0.001 by chi-square test, data not shown). However, BMI is not associated with moist desquamation in our analysis. Another predictive factor for moist desquamation is easy to sweat. It is plausible because sweating usually aggravates symptoms of skin reaction. We use both subjective and objective ways to determine easy to sweat and get more reliable results. Patients with easy to sweat may receive endocrine treatment more frequently. We did an analysis to clarify this concern, and found that there is no significant association between hormone use and easy to sweat in our patients (p value = 0.107 by chi-square test, data not shown). We assumed treatment time in summer may induce more sweating. In Taiwan, a hot and humid season normally lasts from May to September and we chose this period to categorize our patients into two groups for analysis. The results showed that patients received RT during this time frame had a higher incidence of moist desquamation, but the difference did not reach the statistical significance.

The most common location of moist desquamation for our patients is the axillary area. It is not difficult to understand because it is full of sweat glands and skin creases. Only 12 patients developed moist desquamation over the inframammary fold, and it is different from the study of Back *et al.* who found that the inframammary fold is the most common site for moist desquamation [25]. The possible reason is that Chinese women usually have smaller breast size than Caucasian women, and the inframammary fold is not so prominent for our patients. Our results found a high correlation between the risk to develop moist desquamation and the hot spot if the hot spot is located over the axillary or inframammary fold. On the other hand, when moist desquamation occurs at breast and sternal/parasternal area, the majority of them had the hot spot located at these two areas.

This study still has some limitations which need to be addressed. First, we did not use a more scientific method to determine easy to sweat. Indeed, it is difficult to estimate it. We tried to use both self-report (subjective) and clinical observation (objective) ways to eliminate the bias. Second, it is not clear to classify the exact location when the moist desquamation is located over the margin of two areas junction. In this instance, we counted both areas with moist desquamation. Finally, some readers may worry that the contouring of target and breast, and observation of the skin reaction were done by different faculties, and it may raise the interobserver variability. To resolve this concern, the contouring was done by one physician and the skin reaction was supervised by the same physician.

## Conclusion

Our study discovered that the risk for adjuvant RT induced moist desquamation is significantly higher for patients with larger breasts or easy to sweat. The hot spots of skin dose are suggested not to be located at the axillary area and inframammary fold to reduce the incidence of moist desquamation over these two areas.

## Consent

This research was approved and supported by the Zuoying Armed Forces General Hospital (Grant No. ZAFGH-100-22). The reviewers for grant agreed us that IRB approval and patient consent can be waived based on the reasons that (1) the study materials and methods would not impact patient routine treatment, (2) the initial document are from the chart records, and (3) the final data analyzed for publication of this report would not include any identified information of patient.

## Competing interests

There is no any conflict of interest that might constitute an embarrassment to any of the authors.

## Authors’ contributions

LMS participated in design and coordination of study. LMS, EYH, JAL, FYM, GHC, and MJT participated in data acquisition. LMS and EYH contributed to the statistical analysis. LMS, EYH, and JAL contributed to interpretation of data. LMS drafted the article and all other authors helped and finally approved the final manuscript.

## Funding

This study was supported by the Zuoying Armed Forces General Hospital (Grant No. ZAFGH-100-22).
